# An Optimization Model for Expired Drug Recycling Logistics Networks and Government Subsidy Policy Design Based on Tri-level Programming

**DOI:** 10.3390/ijerph120707738

**Published:** 2015-07-09

**Authors:** Hui Huang, Yuyu Li, Bo Huang, Xing Pi

**Affiliations:** 1 College of Economics and Business Administration, Chongqing University, Chongqing 400044, China; E-Mail: huanghui16340@163.com; 2 College of Computer and Information Science, Chongqing Normal University, Chongqing 400047, China; E-Mail: lyyjame@163.com; 3 College of Social Science, Third Military Medical University, Chongqing 400038, China; E-Mail: herryp@163.com

**Keywords:** expired drugs, logistics network, subsidies, multilevel programming, Hybrid Genetic Simulated Annealing Algorithm (HGSAA)

## Abstract

In order to recycle and dispose of all people’s expired drugs, the government should design a subsidy policy to stimulate users to return their expired drugs, and drug-stores should take the responsibility of recycling expired drugs, in other words, to be recycling stations. For this purpose it is necessary for the government to select the right recycling stations and treatment stations to optimize the expired drug recycling logistics network and minimize the total costs of recycling and disposal. This paper establishes a tri-level programming model to study how the government can optimize an expired drug recycling logistics network and the appropriate subsidy policies. Furthermore, a Hybrid Genetic Simulated Annealing Algorithm (HGSAA) is proposed to search for the optimal solution of the model. An experiment is discussed to illustrate the good quality of the recycling logistics network and government subsides obtained by the HGSAA. The HGSAA is proven to have the ability to converge on the global optimal solution, and to act as an effective algorithm for solving the optimization problem of expired drug recycling logistics network and government subsidies.

## 1. Introduction

With the development of the economy, people pay more attention to their health. As a result, families keep more spare drugs in case of emergency, resulting in a dramatic increase in the number of expired drugs. In China, about 78.6 percent of families keep spare drugs, which produce 15,000 tons expired drugs per year [1]. If mistreated, expired drugs are very dangerous, as they can injure people’s health if they carelessly take expired drugs, or badly damage the environment if they are thrown out without the right treatment. Therefore, how to recycle and treat expired drugs at the lowest cost has become an urgent problem that needs to be solved by the government [2].

Although there are lots of studies on recycling expired drugs, most of them are from a medical angle or about the *status quo* of recycling expired drugs [3,4]. Wang, Zhang and Zhang analyzed the dilemma faced by Chinese government in recycling expired drugs, and proposed government’s responsibilities and obligations in this task, and put forward policies for the government [5]. Zhao and Ji analyzed the hazards of expired drugs and the *status quo* of drug recycling management in China, and proposed policies for expired drug disposal [6]. Liu and Qiu compared the effects of taxes and subsidies on expired drug recycling under the assumption that pharmaceutical manufacturing enterprises are responsible for recycling expired drugs, and found that subsidies are more economically reasonable and realistic [7].

There is some research on how to optimize expired drug recycling networks with the purpose of reducing operation costs. Kongar *et al.* proposed a reverse logistics framework that embodies environmental, economical, and physical concerns for end-of-life pharmaceutical products, and provided a radio frequency identification-based information technology infrastructure for the proposed system [8]. Kumar, Dieveney and Dieveney used the DMAIC process to analyze the pharmaceutical supply chain to improve the reverse logistics in a drug recall to avert the possibility of harm to consumers [9]. Narayana, Elias and Pati presented a systemic analysis of the complex interaction of factors affecting the reverse logistics process in a pharmaceutical supply chain, and found that there is strong linkage between the reverse logistics network design and key activities in returns management [10]. Kabir looked into detailed aspects of reverse logistics in the issues that pharmaceutical organizations face, and proposed that addressing the sustainability issue is beneficial to pharmaceutical organizations with end-of life products [11]. Amaro and Barbosa-Póvoa presented a modeling approach for the sequential planning and scheduling of supply chain structures with reverse flows, and applied it to the solution of a real case study of an industrial pharmaceutical supply chain [12].

However, no matter who are the executors of the recycling and disposal of expired drugs, pharmaceutical manufacturing enterprises or drugstores [13,14], and how optimal the expired drugs recycling logistics network is, the operational cost are so high that few executors have any interest in the process [9,15]. Even though the government can force enterprises to recycle and dispose of expired drugs by legislation or regulation [8], they may only recycle or dispose of part of the total volume of expired drugs due to the government’s incomplete supervision. In addition, most of the expired drugs’ owners are average consumers [16], and instead of sending back expired drugs, they may throw them away freely if they must bear the costs of returning expired drugs. Consequently, the result of expired drug recycling is not as good as expected, and it has been suspended in some regions of China [17].

Therefore, the most reasonable solution to the problem of recycling and disposing of expired drugs is that the government becomes the core of recycling expired drugs and drugstores become the executors of recycling expired drugs. In this solution, government takes the responsibility for designing the recycling logistics network to minimize the operation costs of recycling expired drugs, and optimizing the policies for subsidizing residents and drugstores to cover their costs incurred in recycling expired drugs. As a result, all the expired drugs should be recycled at the lowest total cost.

This paper has two main contributions. Firstly, in this paper, the government is the core of expired drug recycling with the responsibility of designing the network, and uses a subsidy policy instead of just legislations or regulations as an incentive mechanism for the residents and drugstores. Secondly, it proposes a tri-level programming model to optimize the recycling logistics network and government subsidy policy, whereby people have the expired drugs, some drugstores are selected to take the responsibility of recycling expired drugs, and parts of the garbage disposal station network are selected to take responsibility for disposing of expired drugs, as a result, all of the expired drugs are recycled and disposed of, while the total costs are minimized.

The remainder of the paper is organized as follows: Section 2 is dedicated to the problem description and notations. The model is established in Section 3. We propose a HGSAA for the model in Section 4. An experiment is used to demonstrate the performance of the HGSAA in Section 5. Finally, conclusions are drawn in Section 6.

## 2. Problem Description and Notations

In order to reduce the family-owned expired drugs’ damage to the environment or threats to people’s health, a regional government decides to establish a recycling mechanism and networks to recycle residents’ expired drugs. The target of the government’s decision-making is recycling all the expired drugs at the lowest cost. Therefore, from *N* alternative drugstores in the region, the government selects *n (n* = 1,2,…,*N)* drugstores as recycling stations to recycle expired drugs from the residents in *I* residential sections. Meanwhile, from *M* alternative waste treatment stations, the government selects *m* (*m* = 1,2,…,*M*) waste treatment stations and upgrades them as expired drug treatment stations to dispose of expired drugs. The fixed costs and operation costs for each recycling station and treatment station are different. Their fixed costs are irrelative to the kind of expired drugs, while their operation costs vary according to the kind of expired drugs. The residents sending expired drugs causes them certain costs, which are irrelative to the kind of expired drugs, while they vary directly with the distance from their homes to the recycling stations and the weight of the expired drugs. To encourage residents to send expired drugs to a recycling station, the government provides a certain amount of subsidies only in accordance with the number of expired drugs sent to the recycling station because the government is unable to know from where the residents send the expired drugs. As a matter of course, if the subsidies from government are more than the cost of sending expired drugs, people are willing to send them, or else, they refuse. Meanwhile the government gives the drugstores a certain amount of subsidies in accordance with the number of every kind of expired drugs recycled by them. As a matter of course, if the subsidies from the government are more than its recycling costs, the drugstore will be willing to be a recycling station, or else, it refuses. The government bears the freight costs of transporting expired drugs from recycling stations to treatment stations, and the fixed and operation costs of treatment stations.

The decision-making process of all participants is as follows: firstly, the government selects *n* drug stores as recycling stations, and *m* waste treatment stations as expired drug treatment stations, and establishes subsidy policies for residents and drugstores, in order to recycle all the expired drugs at the lowest cost. Then, targeting at maximizing the own profits, the selected drugstores decide whether to be a recycling station based on the government’s subsidies and their costs. Finally, targeting at maximizing their own profits, the residents determine whether and where to send expired drugs based on government’s subsidies and their freight cost. The notations used in this paper are as follows:

Φ*_n_*: the set of *n* (*n* = 1,2,…,*N*) recycling stations, Φ*_n_* ⊆ Φ*_N_* and Φ*_n_* ≠ ∅, a decision variable, where Φ*_N_* = {1,2,…,*N*} is the set of all alternative drugstores.

Φ*_m_*: the set of *m* (*m* = 1,2,…,*M*) expired drug treatment stations, Φ*_m_* ⊆ Φ*_M_* and Φ*_m_* ≠ ∅, a decision variable, where Φ*_M_* = {1,2,…,*M*} is the set of all alternative waste treatment stations.

*P_i_*: the number of residents in residential section *i*, *I* = 1,2,…,*I*.

*α_s_*: the average amount of expired drug *s* owned by every resident, *s* = 1,2,…,*S*, where *S* is the drug category number.

*L_ij_*: the distance between residential section *i*, (*i* = 1,2,…,*I*) and alternative drugstore *j*, (*j* = 1,2,…,*N*).

*L_jk_*: the distance between alternative drugstore *j* (*j* = 1,2,…,*N*) and alternative waste treatment station *k*, (*k* = 1,2,…,*M*).

*D_j_*: the decision of the *j^th^* (*j* ∈ Φ*_n_*) selected drugstore on whether to be a recycling station, where the decision variable *D_j_* = 0 means not to be a recycling station, *D_j_* = 1 means to be a recycling station.

*R_ij_*: the decision of residents in residential section *i*, (*i* = 1,2,…,*I*) on whether and where to send expired drugs, *R_ij_* = 0 means sending no expired drugs to a recycling station *j* (*j* ∈ Φ*_n_*), *R_ij_* = 1 means sending expired drugs to one of the recycling stations *j*—a decision variable.

*s_jk_*: the decision on whether to send expired drugs *s* (*s* = 1,2,…,*S*) from recycling station *j* (*j* ∈ Φ*_n_*) to selected treatment station *k* (*k* ∈ Φ*_m_*), *s_jk_* = 0 means sending no expired drugs *s* from recycling station *j* to the selected treatment station *k*, *s_jk_ =* 1 means sending expired drugs *s* from recycling station *j* to selected treatment station *k*—a decision variable.

*β_1_*: the subsidies given to the residents per unit of expired drugs—a decision variable.

*β_2_*: the subsidies given to the recycling stations per unit of expired drugs—a decision variable.

*γ_1_*: the freight fee per expired drug per distance of the residents sending expired drugs.

*γ_2_*: the freight fee per expired drugs per distance of sending expired drugs from the recycling station to the treatment station.

*C_j_*: the fixed costs of alternative drug store *j*, (*j* = 1,2,…,*N*).

*C_k_*: the fixed costs of alternative waste treatment station *k*, (*k* = 1,2,…,*M*).

*C_js_*: the unit operation cost of the recycling station *j* (*j* = 1,2,…,*N*) for expired drug *s*, (*s* = 1,2,…,*S*).

*C_js_*: the unit operation cost of treatment station *k* (*k* = 1,2,…,*M*) for expired drug *s*, (*s* = 1,2,…,*S*).

Among the above notations, Φ*_n_*, Φ*_m_*, *β_1_*, *β_2_*, *R_i_*, *D_j_* and *s_jk_* are decision variables. All of the above information is assumed to be common knowledge for the residents, drugstores and the government.

## 3. Model Development

In the decision-making process of recycling and treating expired drugs, the government is in the leadership, the drugstores and the residents are in the followership, that is, they make their decisions according to the government’s policies. Firstly, the government makes the decisions on the set of recycling stations and treatment stations {Φ*_n_*, Φ*_m_*}, the routes for transporting the expired drugs from recycling stations to treatment stations {*s_jk_*}, and the subsidy policies {*β_1_*, *β_2_*}. Then, the selected drugstores decide whether to be a recycling station {*D_j_*}. Finally, the residents determine whether and where to send expired drugs {*R_i_*}. We will adopt backward induction to obtain their optimal decisions.

In expired drug recycling, the profits of residents in residential section *i* are:

(1) $$\pi_{i}=R_{ij}[(\beta_{1}-\gamma_{1}L_{ij})\sum_{s=1}^{S}\alpha_{s}],j\in \Phi_{n}(D_{j}=1),i=1,2,\ldots ,I.$$

Obviously, the decision target of residents is maximizing their profits and making the maximum profit non-negative. Therefore, the residents are facing the following program, which is the bottom level model in our tri-level model:

(2) $$\underset{R_{ij}}{\max}\;\pi_{i},$$

(3) $$s.t.\underset{R_{ij}}{\max}\;\pi_{i}\geq 0.$$

Solving (2) and (3), we can get the decision of resident *i* as:

(4) $$R_{i}=R_{i}(\Phi_{n}(D_{j}=1),\beta_{1}),$$

that is, the decision of resident *i* is the reaction function of the alternative recycling store *j*’s decision *D_j_* and the government’s subsidy, *β_1_*.

Like all information in this expired drug recycling scenario, the alternative recycling stores know the reaction function of resident *i*, and will make their decisions based on these reaction functions.

Now, we can get the quantity of expired drug *s* (*s* = 1,2,…,*S*) sent to recycling station *j* (*j* ∈ Φ*_n_* (*D_j_* = 1)) is:

(5) $$Q_{js}=D_{j}\sum_{i=1}^{I}\left({\text{R}}_{ij}P_{i}\alpha_{s}\right),j\in \Phi_{n}(D_{j}=1),s=1,2,\ldots ,\text{S}.$$

The profits of recycling station *j* (*j* ∈ Φ*_n_* (*D_j_* = 1)) are:

(6) $$\pi_{j}=D_{j}[\beta_{2}\sum_{s=1}^{S}Q_{is}-C_{j}-\sum_{s=1}^{S}\left(C_{js}Q_{js}\right)],j\in \Phi_{n}(D_{j}=1).$$

The decision target of recycling stations is maximizing their profits and making the maximum profit non-negative. Therefore, the recycling stations are facing the following program, which is the mid-level model in our tri-level model:

(7) $$\underset{D_{j}}{\max}\;\pi_{j},$$

(8) $$s.t.\underset{D_{j}}{\max}\;\pi_{j}\geq 0.$$

Solving (7) and (8), we can get the decision of alternative recycling store *j* as:

(9) $$D_{j}=D_{j}(\beta_{2}),$$

that is, the decision of alternative recycling store *j* is the reaction function of the government’s subsidy *β_1_*.

Like all information in this expired drug recycling scenario, the government knows the reaction function of resident *i* and alternative recycling store *j*, and will make its decision based on these reaction functions.

Now, we can obtain the subsidy given to the residents as:

(10) $$Sub_{I}=\beta_{1}\sum_{i=1}^{I}\sum_{s=1}^{S}\left(R_{ij}P_{i}\alpha_{s}\right).$$

The subsidy given to the recycling stations as:

(11) $$Sub_{J}=\beta_{2}\sum_{j\in \Phi_{n}(D_{j}=1)}\sum_{s=1}^{S}Q_{js}.$$

As the total quantity of expired drug *s* (*s* = 1,2,…,*S*) sent to recycling station *k* (*k* ∈ Φ*_m_*) is:

(12) $$Q_{ks}=\sum_{j\in \Phi_{n}(D_{j}=1)}\left(s_{jk}Q_{js}\right).$$

The total cost of expired drug treatment station *k* is:

(13) $$C_{ts}=\sum_{s=1}^{S}\left(C_{ks}Q_{ks}\right)+C_{k}.$$

The total cost of all expired drug treatment stations is:

(14) $$TC_{ts}=\sum_{k\in \Phi_{m}}C_{ts}.$$

The total freight fee of transporting expired drugs to the treatment stations is:

(15) $$TC_{f}=\gamma_{2}\sum_{j\in \Phi_{n}(D_{j}=1)}\left(s_{jk}Q_{js}L_{jk}\right).$$

Therefore, the total cost borne by government is:

(16) $$TC=Sub_{i}+Sub_{j}+TC_{f}+TC_{ts}.$$

The decision target of government is recycling and treating all the expired drugs at the lowest cost. Therefore, the government is facing the following program, which is the upper level model in our tri-level model:

(17) $$\underset{\Phi_{n},\Phi_{m},\beta_{1},\beta_{2},\left\{s_{jk}\right\}}{\min}TC,$$

(18) $$s.t.\Phi_{n}\in \Phi_{N}=\left\{1,2,\ldots ,N\right\},$$

(19) $$\Phi_{m}\neq \emptyset ,$$

(20) $$\Phi_{m}\subseteq \Phi_{M}=\left\{1,2,\ldots ,M\right\},$$

(21) $$\Phi_{m}\neq \emptyset ,$$

(22) $$\sum_{k\in \Phi_{m}}\sum_{s=1}^{S}Q_{ks}=\sum_{i=1}^{I}\sum_{s=1}^{S}Q_{is}.$$

## 4. The Hybrid Genetic Simulated Annealing Algorithm

This multilevel programming problem is a typical NP-hard problem without a polynomial solution [18]. The genetic algorithm (GA) is widely used for NP-hard problems due to its outstanding capability of globally searching for the optimum. However, due to the GA’s prematurity weakness, which can lead to the optimal solution found by the GA not being the real global optimal solution, many scholars adopt a hybrid genetic algorithm to solve multilevel programming problems [19]. Li *et al.* adopted a hybrid genetic algorithm to solve robust bi-level programming problems [20]. Li *et al.* proposed a hierarchical chaotic quantum-inspired genetic algorithm to solve a nonlinear bi-level programming problem, and verified the effectiveness of their algorithm [21].

Simulated annealing algorithm (SAA) is theoretically able to find the real global optimum, which remedies the shortcomings of the genetic algorithm [22,23]. Therefore, we adopt a hybrid genetic simulated annealing algorithm (HGSAA), whose particularities are converging more rapidly and obtaining the solution more accurately, to solve the tri-level programming of optimizing the recycling logistics network and subsidies for expired drugs.

### 4.1. The Algorithm Thought

The basic thought of the HGSAA in our paper is that GA is developed to rapidly search for an optimal or near-optimal solution among the solution space, and then SAA is utilized to seek a better one on the basis of that solution. Therefore, the disadvantages of genetic algorithms, which are prematurity and weak local searching capability, are effectively avoided, and the global and local search ability of the algorithm is enhanced. As a result, the global optimal solution is found rapidly.

### 4.2. The HGSAA for the Expired Drugs Recycling Logistic Networks

#### 4.2.1. Encoding

We use binary encoding in the HGSAA. If drugstore *i* or waste treatment station *k* are selected, their code will be set as 1; if drug store *i* or waste treatment station *k* are not selected, their code will be set as 0.

#### 4.2.2. Fitness Function

As the objective function is minimizing the government’s total cost, and the fitness function values must be nonnegative, so we define the fitness function as $Fit(\Phi_{n},\Phi_{m})={\left(\frac{1}{2}\right)}^{\log(TC(\Phi_{n},\Phi_{m}))}$, where (Φ*_n_*, Φ*_m_*) is the set of selected recycling stations and treatment stations, and *TC* (Φ*_n_*, Φ*_m_*) is the government’s total cost under this set.

#### 4.2.3. Selection

The selection method in the algorithm is roulette-wheel-selection. The greater the individual fitness value is, the more probable it is that the individual will be selected to be the next parent. The process of selection is as follows: firstly, calculate the selected probability *Pi* of the individual *i*, $P_{i}=Fit_{i}/\sum_{j=1}^{J}Fit_{j}$, where *i* is the set of recycling stations and treatment stations (Φ*_n_*, Φ*_m_*), *j* is the size of the initial population. Then, generate a random number *r* which belongs to [0,1]. Finally, the individual *i* is selected if $\sum_{j=0}^{i-1}P_{j}\leq r\leq \sum_{j=0}^{i}P_{j}$, where *P_0_* = 0.

#### 4.2.4. Crossover and Mutation

Theoretically, the value of both the crossover probability *p_c_* and the mutation probability *p_m_*. ranges from 0 to 1. In common practice, *p_c_* ranges from 0.4 to 0.99, and *p_m_* ranges from 0.001 to 0.1 [24]. In this paper, we use sequencing crossover to exchange the sequence of the operations in the parent chromosomes with the crossover probability *p_c_* = 0.8, and use assignment mutation to change the assignment of a single operation in a single parent with the mutation probability *p_m_* = 0.05.

#### 4.2.5. Simulated Annealing

In the genetic simulated annealing algorithm, through crossover and mutation, parent individuals *p_1_* and *p_2_* generate child individuals *c_1_* and *c_2_*, which are accepted as the individuals of the next population with the probability *P* by calculating their fitness *Fit_p_* and *Fit_c_*. The acceptance probability *P* is shown as follows:

(23) $$P=\{\begin{array}{cc} 1 & Fit_{c}>Fit_{p}\\ \exp\left(\frac{Fit_{c}-Fit_{p}}{t}\right) & Fit_{c}\leq Fit_{p} \end{array}$$

#### 4.2.6. Termination or Convergence Criterion

If the algorithm satisfies the following criteria, then it terminates and outputs the best chromosome, that is, the optimal recycling logistic networks and subsidies:

(i) The fitness value has no change after successive iterations, which means the current solution is the optimal solution.
(ii) The number of iterations reaches the set value, $\frac{\mathrm{lg}({\text{T}}_{\text{e}}{\text{/T}}_{0})}{{\mathrm{lg}}_{\mu }}\times I$, where *I* is the iteration number of the genetic algorithm, *T_0_* is the start temperature, *T_e_* is the stop temperature, and *μ* is the annealing rate.

### 4.3. The Steps of the HGSAA

The steps of the HGSAA are as follows:

Step 1 (Initialization). Get the encoding length, and set the population size *S*, the crossover probability *p_c_*, the mutation probability *p_m_*, the iteration number of the genetic algorithm *I*, the start temperature *T_0_*, the stop temperature *T_e_*, and the annealing rate *μ*.

Step 2. Generate the initial population *p_0_* randomly, then evaluate the fitness function for the current population, and get the current best chromosome.

Step 3. Perform selection, crossover, and mutation operations on the current population to generate an offspring population *p_1_*.

Step 4. Perform a simulated annealing operation on the population *p_1_* to generate a new population *p_2_*, then evaluate the fitness function for *p_2_*, and update the best chromosome.

Step 5. Terminate the process and output the best chromosome when the termination or convergence criterion is satisfied, otherwise, update the temperature and go to step 3 again.

## 5. Experimental Analysis

**Figure 1:**
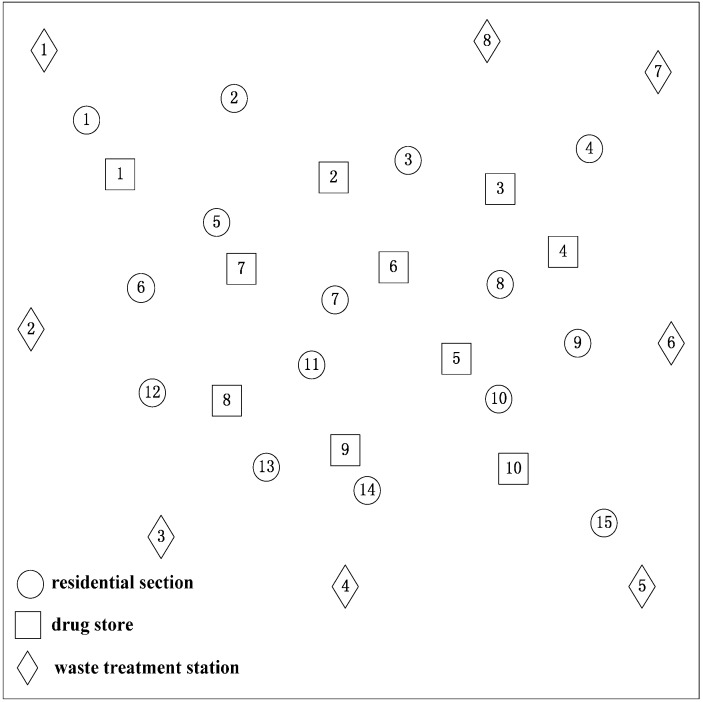
Distribution of nodes in the expired drug recycling logistics network.

In this section, we use an arbitrarily chosen numerical experiment to illuminate our model. The government of one region plans to establish a logistics network and subsidy policy to recycle and treat all the expired drugs owned by the residents in this region. There are 15 residential sections, 10 drugstores (alternative recycling stations) and eight waste treatment stations (alternative expired drug treatment stations), whose location is shown in Figure 1. There are three kinds of expired drugs owned by the residents in this region. The average quantity of each kind of expired drugs in every resident is respectively *α_1_* = 0.014, *α_2_* = 0.02, and *α_3_* = 0.016. The unit freight fee of the residents for sending the expired drugs is *γ_1_* = 4, and that of transporting expired drugs from recycling stations to treatment stations is *γ_2_* = 10. The distance between residential sections and drugstores is shown in Table 1. The distance between drugstores and waste treatment stations is shown in Table 2. The number of residents in every residential section is shown in Table 3. The fixed and operation costs of every alternative recycling station are shown in Table 4 and Table 5, respectively. The fixed and operation costs of every alternative treatment station are shown in Table 6 and Table 7, respectively.

**Table 1:** Distance between residential sections and drugstores.

Drugstore		1	2	3	4	5	6	7	8	9	10
	Residential Section										
1		0.2236	1.6279	2.5495	3.2705	2.8049	2.1523	1.4705	2.1467	3.0232	3.7138
2		0.8732	0.8322	1.7270	2.5129	2.2939	1.5700	1.3509	2.1533	2.7681	3.2450
3		1.9000	0.4123	0.5831	1.4058	1.5529	0.9124	1.5240	2.1745	2.3195	2.4520
4		3.1064	1.6031	0.7071	0.7102	1.6720	1.5207	2.4824	2.9011	2.5807	2.2074
5		0.7826	0.8382	1.7007	2.3454	1.8374	1.1853	0.6519	1.4501	2.1477	2.7586
6		1.1180	1.6401	2.3409	2.7872	1.9503	1.5628	0.5590	0.8488	1.8439	2.7060
7		1.9209	1.1000	1.2728	1.5101	0.6896	0.3905	0.7762	1.0161	1.1045	1.5914
8		2.6000	1.2728	0.7000	0.5886	0.6177	0.6576	1.6500	1.8969	1.5264	1.3647
9		3.3541	2.0518	1.3416	0.6003	0.9009	1.3865	2.3049	2.3393	1.6125	0.9394
10		3.0160	1.9330	1.5001	1.0600	0.4123	1.1220	1.8517	1.7400	0.9604	0.5787
11		2.1260	1.5033	1.6401	1.7281	0.7026	0.7826	0.8846	0.7473	0.7280	1.4151
12		1.9164	2.1915	2.6800	2.9074	1.8847	1.8028	1.0296	0.4415	1.4080	2.3927
13		2.6000	2.3537	2.5239	2.4840	1.3963	1.6771	1.4221	0.6800	0.6083	1.6008
14		3.1341	2.5125	2.3901	2.0983	1.1163	1.7000	1.8868	1.3360	0.3354	0.8860
15		4.3186	3.2757	2.7203	2.0121	1.7492	2.4703	3.1004	2.7496	1.7088	0.8139

**Table 2:** Distance between drugstores and waste treatment stations.

Waste Treatment Station		1	2	3	4	5	6	7	8
	Drugstore								
1		10.4043	15.4829	24.3062	29.3437	34.6468	32.6038	36.6012	14.6986
2		11.9017	16.9664	24.2186	29.2002	33.1436	31.1026	35.1023	13.2231
3		12.8000	17.8474	24.0712	29.0110	32.2349	30.2007	34.2053	12.3628
4		13.3934	18.3933	23.5258	28.4335	31.6328	29.6227	33.6414	11.9073
5		12.5614	17.5001	22.8127	27.7645	32.5010	30.5177	34.5490	12.9131
6		12.1648	17.1643	23.4307	28.4004	32.8623	30.8526	34.8707	13.1104
7		11.1720	16.1611	23.3009	28.3128	33.8595	31.8539	35.8736	14.1107
8		11.1811	16.0812	22.5004	27.5154	33.9200	31.9465	35.9813	14.3533
9		12.1655	17.0144	22.0250	27.0000	33.0038	31.0522	35.0964	13.6015
10		13.1606	18.0156	22.0455	26.9685	32.0047	30.0570	34.1031	12.6650

**Table 3:** Population of residential section.

Residential Section	1	2	3	4	5	6	7	8	9	10	11	12	13	14	15
Population	1354	1203	1532	1610	1437	1180	1875	2539	1649	1293	1560	2160	2312	1480	1727

**Table 4:** Fixed cost of drugstores recycling expired drugs.

Drugstore	1	2	3	4	5	6	7	8	9	10
Fixed Cost	100	110	120	130	90	90	100	110	100	90

**Table 5:** Operation costs of drugstores recycling each kind of expired drug.

Drugstore		1	2	3	4	5	6	7	8	9	10
	Operation Cost										
1		1.9	2	2.1	1.9	2	2.1	2	2.1	1.9	2
2		2	2.1	2.2	2	2	2.2	2.2	2.2	2.2	2.1
3		2	2.2	2.1	2.2	2.2	2	2	2	2	2.2

**Table 6:** Fixed costs of waste treatment stations treating expired drugs.

Waste Treatment Station	1	2	3	4	5	6	7	8
Fixed Cost	6400	6000	7000	6500	8000	5800	6800	7500

**Table 7:** Operation costs of waste treatment stations treating each kind of expired drug.

Waste Treatment Station		1	2	3	4	5	6	7	8
	Operation Cost								
1		700	1000	900	800	600	1000	700	700
2		1200	1400	1200	1000	1000	1800	1000	800
3		1000	1100	700	1000	900	1300	900	950

**Figure 2:**
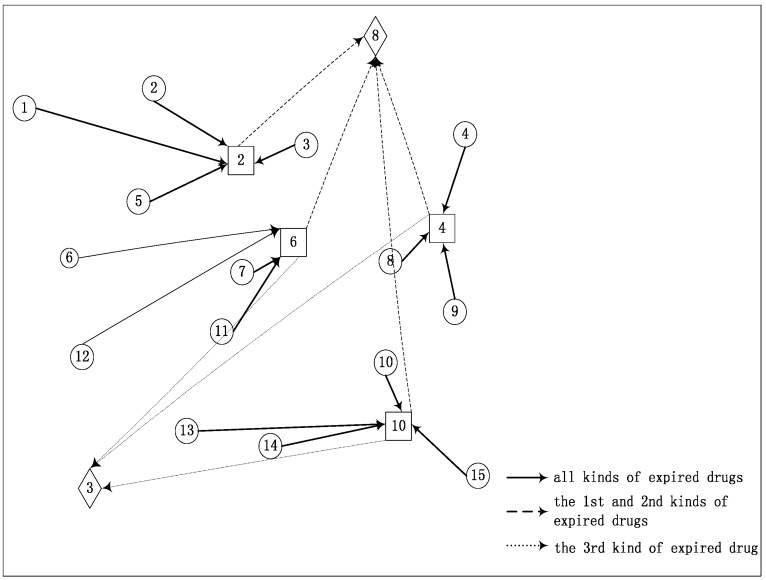
The optimal recycling logistics network and the flow of expired drugs.

The parameters in our HGSAA are set as follows: the population size *S* = 100, the crossover probability *p_c_* = 0.8, the mutation probability *p_m_* = 0.05, the iteration number of the genetic algorithm *I* = 10, the start temperature *T_0_* = 100, the stop temperature *T_e_* = 1, the annealing rate *μ* = 0.95. The result of HGSAA is shown in Table 8 and Figure 2. From Table 8 and Figure 2, we can find that the HGSAA found the optimal expired drugs recycling logistics networks and the optimal government’s subsidy policies. Thus, it can be proved that the HGSAA is an effective algorithm for solving the optimization problem of recycling expired drugs.

**Table 8:** The HGSAA result.

Optimal Chromosome	Optimal Recycling Logistics Networks	Optimal Subsidy Policies	The Final Number of Iterations	Optimal Fitness Function Value	The Minimum Government Total Cost Got by the HGSAA
010101000100100001	final recycling station: 2,4,6,10 final treatment station: 3,8	*β_1_* = 7.2112 *β_2_* = 2.9918	80	6.2963e-05	1.1498e+06

## 6. Conclusions

In this paper, we have proposed a tri-level programming model to study how the government can design recycling logistics networks and subsidy policies to recycle and treat all the expired drugs owned by the residents at the lowest cost. In addition, combining the rapid global searching ability of a GA and the local searching ability of SAA, we proposed a HGSAA to search for the optimal solution of the model. Finally, an experiment was given to demonstrate the good performance of the HGSAA. It is found that the government can encourage the residents and drugstores to participate in expired drug recycling by optimizing the subsidy policies for them, and minimize the total costs of expired drug recycling by optimizing the expired drug recycling logistic network. As a result, all the expired drugs are recycled at the lowest total cost. Besides, it is proved that the HGSAA has the ability to converge on the global optimal solution rapidly and is an effective algorithm for solving the optimization problem of expired drug recycling and treatment policy for the government supply chain.
